# NYU-EDA in modelling the effect of COVID-19 on patient volumes in a Finnish emergency department

**DOI:** 10.1186/s12873-020-00392-1

**Published:** 2020-12-11

**Authors:** Jalmari Tuominen, Ville Hällberg, Niku Oksala, Ari Palomäki, Timo Lukkarinen, Antti Roine

**Affiliations:** 1 Faculty of Medicine and Life Sciences, Tampere University, Tampere, Finland; 2 Emergency Department, Kanta-Häme Central Hospital, Hämeenlinna, Finland; 3 Division of Vascular Surgery, Tampere University Hospital, Tampere, Finland; 4 City of Helsinki, Social Services and Health Care, Helsinki, Finland

**Keywords:** COVID-19, SARS-COV-19, Emergency department, NYU-EDA

## Abstract

**Background:**

Emergency departments (EDs) worldwide have been in the epicentre of the novel coronavirus disease (COVID-19). However, the impact of the pandemic and national emergency measures on the number of non-COVID-19 presentations and the assessed acuity of those presentations remain uncertain.

**Methods:**

We acquired a retrospective cohort containing all ED visits in a Finnish secondary care hospital during years 2018, 2019 and 2020. We compared the number of presentations in 2020 during the national state of emergency, i.e. from March 16 to June 11, with numbers from 2018 and 2019. Presentations were stratified using localized New York University Emergency Department Algorithm (NYU-EDA) to evaluate changes in presentations with different acuity levels.

**Results:**

A total of 27,526 presentations were observed. Compared to previous two years, total daily presentations were reduced by 23% (from 113 to 87, *p* < .001). In NYU-EDA classes, Non-Emergent visits were reduced the most by 42% (from 18 to 10, *p* < .001). Emergent presentations were reduced by 19 to 28% depending on the subgroup (*p* < .001). Number of injuries were reduced by 25% (from 27 to 20, p < .001). The NYU-EDA distribution changed statistically significantly with 4% point reduction in Non-Emergent visits (from 16 to 12%, *p* < .001) and 0.9% point increase in Alcohol-related visits (from 1.6 to 2.5%, p < .001).

**Conclusions:**

We observed a significant reduction in total ED visits in the course of national state of emergency. Presentations were reduced in most of the NYU-EDA groups irrespective of the assessed acuity. A compensatory increase in presentations was not observed in the course of the 3 month lockdown. This implies either reduction in overall morbidity caused by decreased societal activity or widespread unwillingness to seek required medical advice.

## Background

COVID-19 is a an infectious disease caused by severe acute respiratory syndrome virus 2 (SARS-CoV-2) [1]. The spectrum of the disease ranges from asymptomatic to severe, sometimes requiring prolonged treatment in intensive care unit (ICU). The estimated fatality rate is approximately 1.1% and the proportion of patients that require hospitalization range from 1.1 to 18.4% with hospitalization and mortality rates sharply increasing in older population [2]. COVID-19 puts serious strain on ICU and inpatient capacity of healthcare systems and has been mitigated by suspending non-urgent care [3, 4]. Emergency care however has remained fully operational as public health measures against COVID-19 are unlikely to have major effect on incidence of non-infectious emergencies.

COVID-19 is the third epidemic caused by coronaviruses in the twenty-first century [5]. Serious acute respiratory syndrome (SARS) epidemic emerged in China in 2003 and spread to several countries. Middle East respiratory syndrome virus (MERS) emerged in 2012 in Saudi Arabia and spread to 27 countries [5]. The clinical picture of COVID-19 differs from the previous pandemics by larger proportion of mild cases who may remain active in the society and facilitate the spread of the virus [6]. Even though the scale of previous epidemics has been considerably smaller, the literature from heavily affected areas provides valuable information on patient flow dynamics in the face of an epidemic. Reports from SARS outbreak in Taiwan and Hong Kong showed a decline of the number of ED patients by approximately 30% while the proportions of different patient segments remained largely unchanged [7]. In a Canadian study, SARS outbreak resulted in an overall decrease of ED visits where the reduction was mostly explained by the lower attendance of pediatric patients [8]. On the contrary, SARS epidemic in Singapore resulted in an overall increase of ED presentations by about 30%. The increase was explained by increased number of severe patients brought by ambulance and patients seeking help for respiratory symptoms [9].

Finland has not been spared by the impact of COVID-19. To protect the population from the consequences of a widespread disease, the Government of Finland declared a national state of emergency on March 16, 2020. In practice this meant closing the schools together with rapid introduction of e-learning, closure of nightlife establishments and restaurants, and restricting movement to and from the most infected and densely populated Helsinki capital area to the rest of the country. The state of emergency was lifted on June 16, 2020, but extensive restrictions to certain areas of life (travel, mass gatherings) are still applied.

Reports from emergency department patient flows in COVID-19 era have started to appear [10–14]. The key findings have been an evenly distributed reduction of 30–50% in presentations across patient groups and an increase in relative hospitalization rate, suggesting more serious profile [12]. Most studies stratify the presentations based on organ level diagnosis groups or triage groups. The stratification based on raw administrative data is not trivial since triage classification differs between institutions and practitioners [15]. Direct utilization of ICD-10 classification [16] is also insufficient since many categories contain a wide range of disorders of the organ system ranging from benign to acutely life-threatening. New York University Emergency Department Algorithm (NYU-EDA) was developed to provide a systematic reference for estimation of urgency, preventability and level of care needed for patients with different diagnoses by conducting a full chart review on the Clinical Modification of 10th revision of the International Statistical Classification of Diseases and Related Health Problems (ICD-10-CM) based on 3500 ED records [17]. The reference contains statistical estimation of urgency for different diagnoses (e.g. for ICD-10-CM diagnosis R104 Unspecified chest pain, 68% of are deemed to require ED care and 32% of patients are deemed to be treatable by primary care physician). This validated algorithm could be more descriptive and comparable across institutions in documenting the effect of COVID-19 on our healthcare.

The goal of this study is to assess the effect of state of emergency and COVID-19 pandemic on quality and quantity of presentations in the Emergency Department of a Finnish secondary care hospital. We use NYU-EDA to provide meaningful and intuitive stratification of the arrivals and give a novel perspective into the impact the pandemic had on ED service demand based on visit acuity. To our knowledge, this is the first time NYU-EDA is used in Europe and in COVID-19 context.

## Materials and methods

### Data

Kanta-Häme Central Hospital is a secondary care hospital with catchment population of 171,000 people. The emergency department of Kanta-Häme Central hospital provides acute and critical care from 44,000 to 47,000 patients every year and 16 to 17% of the population uses its services yearly. For the purposes of this investigation, we reviewed all patients presenting at our ED between March 16 and June 11 in 2018, 2019 and 2020. When reporting the results, this period is used unless other specified. Mean from years 2018 and 2019 was used as a reference to evaluate the impact of COVID-19 on ED service demand during year 2020. In addition to evaluating the ED as a whole, we grouped the patients according to NYU-EDA to assess the effect on different levels of presentation acuity. We investigated absolute and proportional changes in the number of daily presentations stratified by NYU-EDA groups and also analyzed changes in proportions of respective groups. Institutional approval for the study was obtained. .

### NYU-EDA and Conversion

Since NYU-EDA is based on clinical modification of International Classification of Disease (ICD-10-CM) and Finnish healthcare providers use standard ICD-10, direct use of the classification algorithm was not possible. For this reason, diagnoses covering 95% of the visits in this study were selected for manual review where equivalency chart for ICD-10-CM and WHO ICD-10 was created with the goal of placing diagnoses to equivalent categories. The full chart is available from authors upon request. Since diagnosis to NYU-EDA conversion was performed to 95% of the presentations, the resulting counts were corrected by a factor of 0.95^− 1^ to reflect the actual number of presentations in our ED.

### Statistical tools

Mann-Whitney U test was performed for daily presentation incidence. The chi-square (χ2) test was used to compare proportions in different NYU-EDA groups. Statistical significance was specified as *P* < .05. Statistical tests were performed using Python 3.7.8.

## Results

**Fig. 1:**
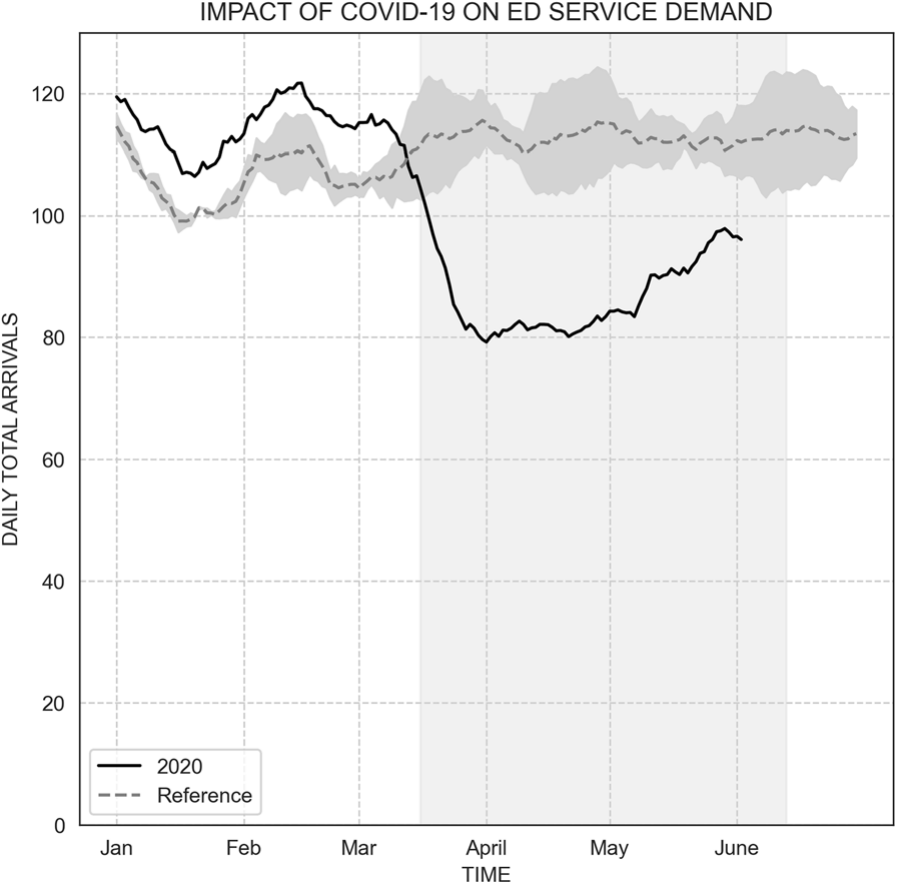
Impact of COVID-19 on daily arrivals. Please note that since 2020 was a leap year, 29th of February was removed from 2020 vector to align the indices. Black solid line = 2020, dark grey area = range in 2018 and 2019, grey dotted line = mean of 2018 and 2019. The light grey box marks the period from March 16 to June 11. Variance is reduced by using a rolling mean with a centered window of 20 days

**Fig. 2:**
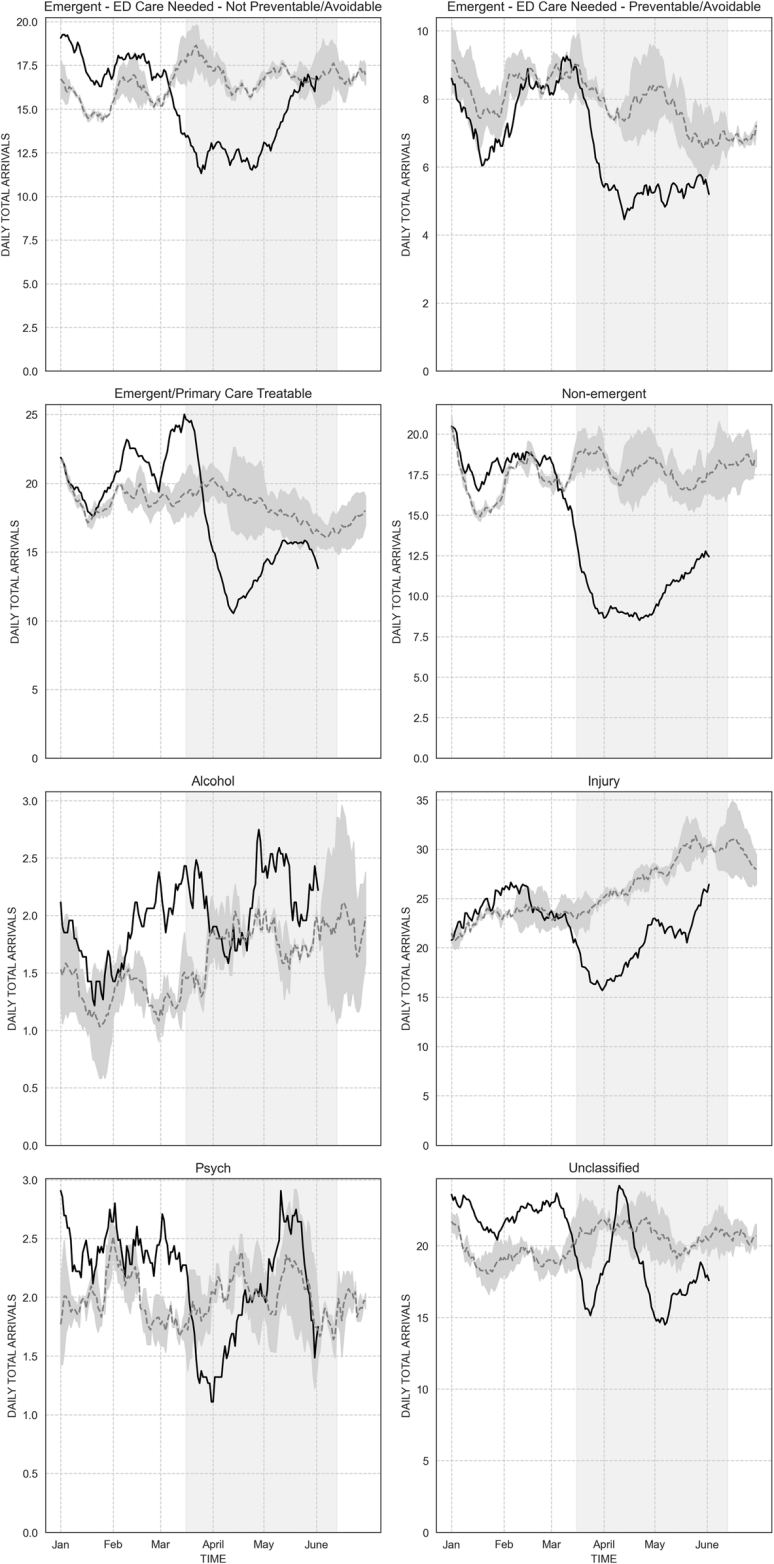
Impact of COVID-19 on daily arrivals as stratified by NYU-EDA groups. Black solid line = 2020, dark grey area = range in 2018 and 2019, grey dotted line = mean of 2018 and 2019. The light grey box marks the period from March 16 to June 11. Variance is reduced by using a rolling mean with a centered window of 20 days

A total of 27,526 episodes of care were included into the analysis. When comparing the mean of 2018 and 2019 with 2020, the total daily number of presentations declined by 23% (from 113 to 87, *p* < .001) (Fig. 1). Non-Emergent visits declined the most with 42% reduction (from 18 to 10, *p* < .001). Injuries, being the most common reason for presentation, were reduced by 25% (from 27 to 20, p < .001). Emergent - ED Care Needed - Not Preventable/Avoidable declined by 19% (from 17 to 13, *p* < .001) having identical proportional reduction with the Emergent/Primary Care Treatable group which also declined by 19% (from 18 to 15, *p* < .001). Emergent – ED Care Needed – Preventable/Avoidable were reduced by 28% (from 7.7 to 5.6, p < .001). Drug related visits were reduced by 29% (from 0.10 to 0.07, *p* = 0.01) whereas alcohol related presentation had a non-significant trend to increase by 23% (from 1.7 to 2.2, *p* = .09 (Table 1, Fig. 2).

**Table 1:** Average daily presentations between March 16 and June 11 stratified by NYU-EDA. Δ2020 describes the difference between 2020 and mean of 2019 and 2018. Arr = daily arrivals, Std = standard deviation, EDCN = Emergency Department Care Needed, ~ = Not, P/A = Preventable/Avoidable, PCT = Primary Care Treatable. Proportional change is provided in the parenthesis

	Study year, Arr ± Std								
	2018		2019		2020		Δ2020	(%)	p
Emergent EDCN ~P/A	16	±3.5	18	±4.1	14	±4.1	−3	(−19)	<.001
Emergent EDCN P/A	7	±2.3	8	±3.0	6	±2.5	−2	(−28)	<.001
Emergent PCT	17	±3.6	20	±4.8	15	±4.6	−4	(−19)	<.001
Non-Emergent	17	±3.8	19	±5.1	10	±3.4	−8	(−42)	<.001
Alcohol	1.6	±1.3	1.9	±1.6	2.2	±1.6	0	(23)	.09
Drug	0.08	±0.3	0.12	±0.3	0.07	±0.3	−0.03	(− 29)	.01
Injury	27	±7.7	27	±6.7	21	±6.8	−7	(−25)	<.001
Psych	2	±1.4	2	±1.5	2	±1.5	0	(−3)	.2
Unclassified	20	±4.9	22	±5.3	18	±5.9	−3	(−15)	<.001
Total	107	±11.4	119	±13.8	87	±12.3	−26	(−23)	<.001

Chi-square showed a statistically significant difference in distributions of Non-Emergent (from 16 to 12%, *p* < 001), alcohol-related (from 1.6 to 2.5%, *p* < .001), Psychiatric (from 1.7 to 2.2%, *p* = .01) and Unclassified (18.4 to 20.4%, p < .001) visits. Injuries constituted the majority of visits but the difference in proportions was not statistically significant (from 24.2 to 23.7%, *p* = .45), which was the case for the other NYU-EDA groups as well (Table 2).

**Table 2:** Total arrivals between March 16 and June 11 stratified by NYU-EDA groups. Δ2020 describes the difference in proportion when comparing year 2020 with pooled visits from 2018 and 2019. EDCN = Emergency Department Care Needed, ~ = Not, P/A = Preventable/Avoidable, PCT = Primary Care Treatable, p.p. = percentage point

	Study year, n (%)							
	2018		2019		2020		Δ2020 in p.p.	p
Emergent EDCN ~P/A	1441	(15)	1554	(15)	1207	(16)	+ 0.7	.14
Emergent EDCN P/A	617	(7)	739	(7)	489	(6)	−0.4	.20
Emergent PCT	1474	(16)	1764	(17)	1309	(17)	+ 0.8	.10
Non-Emergent	1464	(16)	1686	(16)	909	(12)	−4.0	<.001
Alcohol	142	(1.5)	167	(1.6)	190	(2.5)	+ 0.9	<.001
Drug	7	(0)	11	(0)	6	(0)	−0.0	.80
Injury	2394	(25)	2415	(23)	1814	(24)	−0.5	.39
Psych	154	(1.6)	193	(1.8)	168	(2.2)	+ 0.5	.01
Unclassified	1723	(18)	1941	(19)	1564	(20)	+ 2.0	<.001
Total	9416	(100)	10,470	(100)	7656	(100)	0	

## Discussion

We observed significant reduction in the total number of arrivals and in almost every NYU-ED urgency class. The proportional reduction is in line with reports from SARS and MERS pandemics as well as reports from COVID-19 pandemic in other geographies. Somewhat expectedly non emergent visits were almost halved. However, we also observed a significant reduction of 19% in emergent, non-preventable cases. This suggests that acute health concerns have been left untreated due to people’s unwillingness to seek medical advice even if that may have been necessary. This may be explained by the widespread fear of the virus or by the perception by the public that limited healthcare resources should be reserved for COVID-19 patients, causing them to underestimate the importance of their medical problems.

There was almost a 30% reduction in number of preventable emergency department visits in the study period. These visits are characterized by exacerbation of chronic conditions such as diabetes, chronic heart failure and asthma that could be prevented by close monitoring and non-emergent management. This may suggest that the speculated negative effect of the lockdown on the management of chronic conditions did not materialize in increased number of visits. Our setting does not shed light on the definite cause of this phenomenon, but we speculate that it is partly explained by elevated threshold to seek medical attention and successful primary care despite and because of the pandemic. One might also speculate, that the closing of the society by government restrictions might have reduced respiratory tract infections in general and thus exacerbations of some chronic diseases. Respiratory tract infections are known to be associated e.g. with asthma exacerbations and acute cardiac events [18, 19]. It must also be acknowledged that the time period of three months may be insufficient for significant deterioration of chronic conditions to translate into a detectable number of ED visits.

Injury -related presentations decreased by 25%. This falls between the previously reported 50% reduction in a British trauma center and a 16% reduction in emergency surgery in Finnish hospitals [20, 21]. There was not a statistically significant difference in the number of Alcohol related presentations but it was the only group that was increased during the study period. If the observation is indeed real, it. This may be explained by the increased domestic alcohol use as nightlife establishments were closed during the pandemic [22].

The NYU-EDA ICD-10CM chart conversion to ICD-10 was straightforward as the United States version is essentially a more detailed version of the original ICD-10. The utilization of NYU-EDA has clear advantages over ICD-class based -stratification of emergency department visits as many diagnostic classes contain drastically different conditions and without case review -based chart, ICD-diagnosis is difficult to reliably translate into urgency. Although NYU-EDA has not been validated in European datasets, it has been validated in nationwide studies in the United States [23].

The weaknesses of this study are its retrospective nature and limited historical data of two years, the latter of which is caused by a reform in emergency department organization at the institution, preventing direct comparisons with patient flow before year 2018. Certain patient groups such as high-energy polytrauma and ST-elevation myocardial infarctions are treated in a tertiary center in the neighboring hospital district. However, the proportion of these patients is low and we believe the potential bias from that course to be limited. Additionally, the conversion of NYU-EDA ICD-10-CM to ICD-10 chart was limited to 95% of presented codes. However, we consider this acceptable as total conversion of 70,000 ICD-10CM codes would have provided very little additional information on overall demographics of the ED presentations. It is also important to note that our study does not shed light on the underlying causes of the documented reduction, which will be a subject to further investigation. We also highlight that the design of the study and the limited timeframe prevents us from making conclusions on the effect of COVID-19 pandemic on long-term mortality and morbidity of non-COVID patients.

## Conclusions

COVID-19 and the state of emergency significantly affected the number of visits in a Finnish emergency department. Both emergent and non-emergent visits were significantly reduced. Whether this will materialize as a delayed increase in overall morbidity will be an interesting subject for later investigation. The lessons from earlier SARS and MERS pandemics seem to apply to ED patient flow dynamics during COVID-19 pandemic.

## Data Availability

The datasets used and/or analysed during the current study are available from the corresponding author on reasonable request.

## Abbreviations

SARS-CoV-2: Severe Acute Respiratory Syndrome Virus 2
SARS: Serious Acute Respiratory Syndrome
MERS: Middle East respiratory syndrome virus
NYU-EDA: New York University Emergency Department Algorithm
ICD-10-CM: International Statistical Classification of Diseases and Related Health Problems Clinical Modification
ED: Emergency Department
EDCN: Emergency Department Care Needed
PCT: Primary Care Treatable
